# 15. Cluster Headache

**DOI:** 10.1111/papr.70050

**Published:** 2025-05-28

**Authors:** Casper S. Lansbergen, Rolf Fronczek, Leopoldine A. Wilbrink, Steven P. Cohen, Cecile C. de Vos, Frank J. P. M. Huygen

**Affiliations:** ^1^ Department of Anesthesiology, Center for Pain Medicine Erasmus MC University Medical Center Rotterdam the Netherlands; ^2^ Department of Neurology Leiden University Medical Center Leiden the Netherlands; ^3^ Department of Neurology Zuyderland Medical Center Heerlen the Netherlands; ^4^ Department of Anesthesiology, Neurology, Physical Medicine & Rehabilitation, Psychiatry and Neurological Surgery Northwestern University Feinberg School of Medicine Chicago Illinois USA; ^5^ Department of Anesthesiology and Physical Medicine & Rehabilitation, Walter Reed National Military Medical Center Uniformed Services University of the Health Sciences Bethesda Maryland USA

**Keywords:** cluster headache, evidence‐based medicine, interventional treatment, medically intractable chronic cluster headache, neurostimulation

## Abstract

**Introduction:**

Cluster headache is a rare primary headache disorder characterized by excruciating unilateral pain around the eye, lasting between 15 and 180 min, accompanied by ipsilateral cranial autonomic symptoms. Cluster headache is classified into two forms: episodic and chronic, with chronic cluster headache defined by pain‐free intervals of less than 3 months between bouts. Both drug‐based and invasive treatments are available for abortive and preventive purposes. Treatment selection depends on individual efficacy and tolerance, with invasive options considered when pharmacological treatments prove ineffective.

**Methods:**

This narrative review summarizes the literature on common practice and the evidence in the treatment of cluster headache.

**Results:**

Oxygen therapy and subcutaneous sumatriptan are the most effective abortive treatments for cluster headache. Oral corticosteroid tapering regimens can be used as bridging therapy. Verapamil, lithium, topiramate, and CGRP antagonists are potential preventive medication options.

Greater occipital nerve (GON) injections and radiofrequency (RF) therapy can be used as preventive treatments, though their effects are often temporary. For refractory chronic cluster headache, occipital nerve stimulation (ONS) has proven to be effective. Deep brain stimulation (DBS) may also be considered if all other treatments have failed.

**Conclusions:**

The management of cluster headache is complex due to the variable efficacy of treatments across different patients and limited evidence.

## 1 Introduction

This article on Cluster Headache is part of the “Evidence‐Based Interventional Pain Medicine According to Clinical Diagnoses” series [1].

Cluster headache is a debilitating primary headache disorder known for its characteristic unilateral, intolerable pain around the eye, accompanied by ipsilateral cranial autonomic symptoms. These rapid‐onset circannual and circadian headaches manifest in bouts of 1 to 8 attacks per day, with each attack lasting between 15 min and 3 h. Cluster headache is divided into two different forms, episodic and chronic. The latter is defined based on the duration of pain‐free intervals between bouts, which is less than 3 months in chronic cluster headache [2].

The prevalence of cluster headache is about 1 in 1000 individuals, with a male‐to‐female ratio of 4:1 [3]. However, recent literature indicates that the male‐to‐female ratio may be smaller (2:1), though males are still disproportionately affected, especially compared to the strong female‐to‐male predominance in other headache disorders. This may be due in part to women being more likely to be misdiagnosed [4, 5, 6]. Moreover, chronic cluster headache seems to be more prevalent in women than in men. The onset of cluster headache usually occurs before the age of 50 years, with a peak prevalence between 20 and 29 [5]. Approximately 10% to 15% of cluster headache patients experience chronic cluster headaches [3].

The pathophysiology of cluster headache involves complex changes in the central and peripheral nervous system, including activation of the hypothalamus, the trigeminovascular system, and the autonomic nervous system. A comprehensive understanding of these phenomena is lacking. The identification of sleep as a trigger, combined with the circadian nature of the condition, has led to the hypothesis that the hypothalamus plays a fundamental role in the pathogenesis of cluster headache [7].

Cluster headache has a complex genetic component. The results of two independent genome‐wide association studies (GWASs) in European cohort populations revealed the existence of four genetically predisposed loci. The identified gene sites were DUSP10, MERTK, SATB2, and FHL5 [8, 9]. Two of the loci were replicated in an Asian cohort, with an additional locus reported in the gene CAPN2 [10]. A meta‐analysis, which included these studies, confirmed the four previously found loci along with three others in the European cohort. The loci identified in the Asian cohort were replicated in this study, and an association was identified with a nearby locus in the European ancestry meta‐analysis [11]. This may explain the observed interethnic differences and warrants further studies investigating genotype–phenotype associations.

Regarding lifestyle habits and potential risk factors, cluster headache has been associated with smoking, head trauma, and a family history of headaches [12]. A recent genetic meta‐analysis indicated a causal relationship between smoking and cluster headache [11]. For illicit drug use, a causal relationship has not been confirmed, but it is more prevalent in patients with cluster headache, particularly among men [13, 14]. The findings related to alcohol and other substance use disorders are inconclusive, especially in light of the high rate of substance use disorders in headache conditions in general [15, 16]. Other documented triggering factors for attacks include sleep, stress, high altitude, and changes in weather conditions.

Although sleep is recognized as a potential trigger for cluster headaches during bouts, the precise association between cluster headaches and sleep remains unclear. The pattern of attacks that are synchronous with circadian rhythm appears to depend on hypothalamic involvement. Melatonin secretion and associated autonomic dysfunction are also involved in the chronobiology of cluster headache [7]. The nocturnal nature of these attacks often leads to sleep disturbances, which can cause sleep deprivation and exacerbate other sleep disorders. However, it is essential to note that establishing a direct causal relationship has proved challenging [17].

Although cluster headache is a relatively rare form of headache, it is associated with a disproportionately high level of use of healthcare and social services. A significant proportion of individuals with chronic cluster headache—around 94%—report experiencing limitations in their personal and/or professional lives during attack periods. Moreover, 15% of these patients still reported limitations in their general daily activities despite being in remission. Loss of employment due to cluster headache was reported in 21% of patients. This results in a significant economic burden, with a mean total annual cost per patient estimated at €6321 for patients with episodic cluster headache and €20.967 for patients with chronic cluster headache [18]. Comorbidities such as depression, anxiety, and suicidality are strongly associated with cluster headache, highlighting the significant burden of the disease and the need for effective treatment [19, 20, 21].

Currently, the treatment of cluster headache is mostly based on expert opinion and off‐label treatments, as the quality of evidence is limited. This review aims to provide a comprehensive overview of the current state of science and practice for the treatment of cluster headache.

## 2 Methodology

This narrative review on cluster headache is an update of the 2009 article published in the series “Evidence‐based Interventional Pain Medicine According to Clinical Diagnoses” [1].

The primary purpose of this article is to provide a review of the current literature on treatment, with the aim of providing guidance for daily practice. The last literature review was performed in December 2023. We used the keywords “cluster headache” in combination with specific treatment modalities. The terminology for the pharmacological treatment included: “oxygen therapy”, “triptans”, “sumatriptan” “ergotamine”, “lidocaine”, “ketamine”, “Somatostatin analogues”, “corticosteroids”, “frovatriptan”, “verapamil”, “lithium”, “topiramate”, “CGRP antagonists”, “melatonin”, “gabapentin” and “pregabalin”. The terminology for the interventional treatment section included: “greater occipital nerve injections”, “GON‐injections”, “radiofrequency therapy”, “pulsed radiofrequency therapy”, “non‐invasive vagus nerve stimulation”, “sphenopalatine ganglion stimulation”, “occipital nerve stimulation”, “deep brain stimulation” and “spinal cord stimulation”. Additionally, the reference lists of reviewed articles were examined to identify any further relevant studies not captured in the initial search. Given the limited availability of randomized controlled trials (RCTs), lower‐level evidence such as observational studies, case series, and expert opinions was included. Due to the nature of the evidence, which largely consists of case reports, case series, and observational studies, a formal quality appraisal was not conducted. To provide additional insight into the available evidence, we have included an overview of recent systematic reviews and meta‐analyses on interventional treatments in the Appendix A, where the most substantial findings are discussed.

## 3 Diagnosis

### 3.1 History

Cluster headache is diagnosed primarily based on the patient's symptoms and the exclusion of secondary headache etiologies. The diagnostic criteria for cluster headache are defined in the International Classification of Headache Disorders, Third Edition (ICHD‐3; Table 1) [2].

**TABLE 1:** Diagnostic criteria for cluster headache according to the International Classification for Headache Disorders—Third Edition (ICHD‐3) [2].

At least five attacks fulfilling criteria B–D
B Severe or very severe unilateral orbital, supraorbital and/or temporal pain lasting 15–180 min (when untreated)
C Either or both of the following:
at least one of autonomic symptoms or signs (see also Table 3), ipsilateral to the headache:
2 a sense of restlessness or agitation
D Occurring with a frequency between one every other day and 8 per day
E Not better accounted for by another ICHD‐3 diagnosis.
Episodic cluster headache: Cluster headache occurs in episodes with at least two cluster episodes lasting from 7 days to 1 year (untreated), and separated by pain‐free periods of ≥ 3 months.
Chronic cluster headache: Cluster headache has persisted for at least 1 year, without a remission period or with a remission period lasting < 3 months.

A typical cluster headache attack consists of severe, unilateral, sharp pain in the (supra)orbital and/or temporal region. It is often accompanied by a sense of restlessness and/or ipsilateral autonomic dysregulation, most commonly a red, tearing eye, nasal congestion, and/or rhinorrhea [2]. Although one side is usually affected, there are cases in which the painful side may alternate between attacks, sometimes after local treatment of one side.

In 85% of the patient population, a prodromal phase precedes the cluster headache attack, occurring 10 to 20 min before onset. During this phase, more than two‐thirds of patients report dull pain in the (supra)orbital and/or temporal areas, while half report painless local autonomic symptoms such as lacrimation, ptosis, and nasal congestion [22].

Once an actual attack starts, it can last from 15 to 180 min. On average, patients have about four attacks a day, though this can vary between one and eight attacks. The attacks often occur at night, waking the patient from sleep, and may follow a circadian or circannual rhythm. The clustering of the attacks into periods is called bouts. Based on the difference in pain‐free periods between these bouts, two subtypes are distinguished: episodic and chronic cluster headache. Episodic cluster headache is characterized by recurrent bouts that transpire over periods of 7 days to a year, interspersed with pain‐free periods of at least 3 months. In contrast, chronic cluster headache is diagnosed when there is no remission for more than 1 year or when the attack‐free period is less than 3 months [2]. In chronic cluster headache patients, there is a subgroup of patients who do not respond or cannot tolerate at least three different pharmacological treatments (Table 2). This condition is referred to as Medically Intractable Chronic Cluster Headache (MICCH) [23]. The European Headache Federation refers to this similarly defined subtype as refractory chronic cluster headache (rCCH) [24].

**TABLE 2:** Conditions for defining intractable cluster headache [23].

Failed adequate trials of regulatory approved and conventional treatments according to local and national guidelines
*Adequate trial*
Appropriate dose Appropriate length of treatment Medication overuse headache symptoms were considered and ruled out
*Failed*
No therapeutic (or an unsatisfactory) response Intolerable side effects Contraindications to use
In cluster headache, failure of at least four classes of medications with two including numbers 1–3
Verapamil
2 Lithium
3 Methysergide
4 Melatonin
5 Topiramate
6 Gabapentin

Some countries recognize a clinical condition known as “cluster storm”. This condition is characterized by the occurrence of multiple cluster headache attacks in short succession over multiple days, with each attack beginning shortly after the previous one. Despite its recognition, there is an absence of high‐quality peer‐reviewed literature on the characteristics or treatment of this condition.

### 3.2 Physical Examination

Neurological examination of patients with cluster headaches typically reveals no abnormalities. During a cluster headache attack, a number of autonomic features may be observed, which paradoxically tend to decrease along with pain on the ipsilateral side. These are illustrated in Table 3.

**TABLE 3:** Autonomic characteristics of cluster headache [25].

*Ipsilateral to site of pain*
Lacrimation or conjunctival injection Rhinorrhea or nasal congestion Cranial and/or facial sweating Miosis and/or ptosis Edema of the eyelid or orofacial tissues (including gingiva and palate) Facial flushing or pallor

### 3.3 Additional Tests

The diagnosis of cluster headache is usually based on a comprehensive assessment of the patient's medical history. According to the established criteria, this diagnosis should rule out the possibility of underlying structural disorders, making further investigation unnecessary. However, differentiating between primary cluster headaches and secondary headaches with similar features can be challenging. In such cases, brain imaging, particularly MRI, may help exclude secondary causes. Debate continues over the optimal timing of scans and the necessity of a pituitary protocol [26].

The current literature indicates that MRI has potential diagnostic value in cases where the presentation does not meet ICHD‐3 criteria [26]. A routine MRI is therefore not recommended in patients under the age of 40 years in the absence of specific evidence of structural abnormalities or an atypical presentation. It should also be noted that while MRI can be used to exclude secondary causes of cluster headaches, it can also lead to the discovery of unexpected secondary findings. This may lead to unnecessary follow‐up and treatment [27].

### 3.4 Differential Diagnosis

Cluster headache is a primary headache disorder belonging to the category of trigeminal autonomic cephalalgias (TACs). The TACs are defined by the presence of shared clinical features, namely a unilateral headache in combination with ipsilateral cranial parasympathetic autonomic symptoms. The remaining TACs include short‐lasting unilateral neuralgiform headache attacks (SUNCT/SUNA), paroxysmal hemicrania, and hemicrania continua [2]. Although cluster headache is a relatively rare primary headache condition, it is the most prevalent TAC [3]. Despite considerable overlap in clinical presentation between these different conditions, differentiation can be achieved by evaluating the frequency and duration of attacks (Table 4). Both proximal hemicrania and hemicrania continua can be effectively treated with indomethacin, differentiating them from the other TACs [2].

**TABLE 4:** Differentiating characteristic of trigeminal autonomic cephalalgias [2].

Headache	Typical attack frequency	Typical attack duration (if untreated)	Response to indomethacin	Response to oxygen therapy
Cluster headache	Between once every other day to up to 8 per day	15 to 180 min	No	Yes
Paroxysmal hemicrania	> 5 times per day	2 to 30 min	Yes	No
Short‐lasting unilateral neuralgiform headache attacks (SUNCT/SUNA)	At least once a day	1 to 600 s	No	No
Hemicrania continua	Remitting/unremitting	Remitting/unremitting	Yes	No

In addition to TACs, several other conditions should be considered in the differential diagnosis because of the overlap in presentation with cluster headache. Migraines can mimic cluster headache but typically last longer and more often include nausea, vomiting, and aura. Trigeminal neuralgia presents with brief, electric shock‐like pain, often triggered by touch, but typically lacks autonomic symptoms [2]. Acute angle‐closure glaucoma can cause unilateral orbital pain with blurred vision, redness of the eye, and elevated intraocular pressure, distinguishing it from cluster headache [28]. Finally, hypnic headache, which occurs during sleep, typically lacks the autonomic features and severe intensity of cluster headache [2].

## 4 Treatment Options

### 4.1 Pharmacological Management

In recent years, considerable progress has been made in the treatment of cluster headache, including the use of neuromodulation. In the context of cluster headache treatment, a clear distinction is made between three main categories of treatment: symptomatic/abortive, bridging, and preventive/prophylactic. The majority of patients receive both abortive and prophylactic treatment. Abortive treatments are primarily designed to relieve symptoms and shorten the duration of attacks, whereas preventive/prophylactic treatments are intended to decrease the attack frequency. Bridging therapy serves as an interim solution, providing coverage until prophylactic treatment is fully effective. It should be noted that the symptomatic/abortive treatment for both episodic and chronic cluster headache is similar, whereas the prophylactic approach differs.

#### 4.1.1 Symptomatic/Abortive Therapy

An effective abortive therapy should ideally have high bioavailability and provide immediate, substantial relief, preferably with minimal side effects. For these reasons, a rapid form of administration, such as inhalation, nasal, or subcutaneous injection, is often preferred to oral administration.

##### 4.1.1.1 Oxygen Therapy

One of the most effective abortive treatments for an acute cluster headache attack is inhalation of 100% oxygen at a flow rate of 7–12 L/min or more for 15 to 30 min using a non‐rebreathing face mask [29, 30, 31]. The use of demand‐valve oxygen masks and higher flow rates remains a subject of controversy [32, 33]. Although oxygen inhalation can be started at any time during an attack, immediate administration is recommended. This treatment carries a low risk of oxygen toxicity and no serious side effects and can be easily used in combination with sumatriptan [31, 34]. Although rebound headache has been described in the context of oxygen therapy, it is rare [35, 36].

Oxygen therapy can also be inconvenient because oxygen cylinders must be carried, which may limit patient mobility. The use of oxygen therapy may be contraindicated in patients with severe chronic obstructive pulmonary disease because oxygen supplementation reduces respiratory drive, causing hypercapnia [37]. However, oxygen therapy may still be considered for active smokers with appropriate instruction and guidance [31].

##### 4.1.1.2 Triptans

Subcutaneous administration of sumatriptan (5‐HT_1__B/D_‐agonist) 6 mg has been shown to provide significant pain relief and is currently considered the most effective treatment for acute cluster headache attacks [38, 39]. According to official guidelines, the maximum recommended dosing frequency is twice daily. However, due to the high frequency of attacks, this amount is often exceeded by patients, and expert opinion consensus accepts up to eight doses per day. It is noteworthy that no significant side effects have been reported with prolonged and more frequent use of sumatriptan [40, 41]. Nevertheless, caution is advised, especially in patients with certain cardiovascular disease, as triptans are vasoconstrictive and therefore may be contraindicated.

For patients intolerant to injections, the use of sumatriptan 20 mg nasal spray or zolmitriptan 10 mg nasal spray has been shown to be an effective method for abortive treatment [42, 43, 44, 45]. Nevertheless, non‐subcutaneous administration of triptans is only advised in patients with relatively long attacks, exceeding 1 h, because of the longer resorption time by the mucosa. Therefore, oral triptans are generally not indicated for the acute treatment of cluster headaches given their slow duration of onset.

##### 4.1.1.3 Others

Ergotamine derivatives can be administered by various routes, such as aerosol spray, suppository, or subcutaneous injection, to achieve a rapid increase in plasma concentration. To prevent recurrent nocturnal attacks, a dose of 2 mg before bedtime can be effective [46]. Although it was one of the earliest treatments shown to be effective for cluster headache, ergotamine is now rarely used in any type of headache disorder because of serious side effects, including myocardial infarction and limb ischemia [47, 48].

Lidocaine has been studied in open‐label trials for cluster headache treatment, using either intranasal drops or sprays of 4% lidocaine or a cotton swab soaked in 10% lidocaine applied to the posterior nasal cavity, where the sphenopalatine ganglion (SPG) is located [49, 50, 51, 52]. Because of its limited efficacy, lidocaine is only recommended as a secondary option when standard treatments like oxygen therapy and triptans are ineffective or poorly tolerated by patients. Lidocaine has also been explored for intravenous administration as a transitional treatment in several refractory headache cohorts, which have included some cluster headache cases. However, due to the limited number of cluster headache patients in these studies, no substantial data is available to recommend intravenous lidocaine as a primary treatment option [53, 54].

Somatostatin analogues, such as octreotide, are administered subcutaneously in the treatment of cluster headache patients because of their longer half‐life [55]. The normalization in somatostatin levels inhibits neuropeptides that may be involved in pathogenesis, such as calcitonin gene‐related peptide (CGRP) and vasoactive intestinal polypeptide (VIP) [56].

Ketamine, an NMDA receptor antagonist, is widely used for chronic pain and headache treatment [57]. In cluster headache, intravenous ketamine (0.5 mg/kg) has been evaluated in three case series, with or without 3000 mg of magnesium, which also blocks NMDA receptor sites [58, 59, 60]. In patients with episodic cluster headaches, all attacks were aborted [58]. Among chronic cluster headache patients (mostly refractory), the majority experienced a reduction in headache intensity and/or frequency by more than 50% [58, 59, 60]. Notably, the effects persisted for weeks to even months (up to 18 months), suggesting that ketamine may also have potential as a transitional or even preventive treatment.

Intranasal ketamine was also tested in an open‐label pilot study of chronic cluster headache patients, where 15 mg doses were administered every 6 min, up to five doses. A ≥ 50% reduction in pain was achieved in 59% of patients within 30 min [61]. However, the study lacked comparison with placebo, other acute treatments, or SPG therapy, warranting further controlled trials [62].

#### 4.1.2 Transitional Therapy

Transitional therapy, also known as bridging therapy, generally provides rapid‐onset benefit lasting from weeks to months, until preventive therapy can take effect. During this time, preventive treatment can be titrated gradually and optimized, promoting therapeutic benefit while minimizing side effects. Sometimes, transitional treatment can be used to treat short bouts without the need to initiate preventive medication.

##### 4.1.2.1 Oral Corticosteroids

Corticosteroids are effective and have a rapid onset of action with a relatively short duration, making them well suited for transitional therapy [63, 64]. Corticosteroids can be administered orally, intravenously, or by injection at the greater (and lesser) occipital nerves. Despite the long‐standing use of corticosteroids in the treatment of cluster headache, consensus has not yet been reached on the optimal treatment regimen. A major reason for this disagreement is the challenging balance between the efficacy of corticosteroids and their potential long‐term side effects, including weight gain, osteoporosis, avascular necrosis, and adrenal insufficiency.

Oral corticosteroid treatment is offered in various schedules, usually lasting between 2 to 3 weeks. A commonly used schedule begins with an initial dosage of 100 mg of prednisone (or equivalent corticosteroid) daily for a period of 5 days. The dose is then reduced by 20 mg every 3 days [64]. Additionally, alternative regimens have been suggested, such as a starting dose of 60 mg with a reduction of 5 mg every 5 days. Intravenous treatment is usually performed with a bolus of methylprednisolone, with varying doses and durations [65, 66]. However, this requires the need for hospitalization, making it impractical in many circumstances. The lack of comparative‐effectiveness studies evaluating administration and dosage makes this a promising target for future research.

##### 4.1.2.2 Frovatriptan

Frovatriptan, a selective 5‐HT_1B/D_‐agonist, has yet to be proven as definitively effective for treating episodic cluster headaches, though anecdotal evidence is promising [67]. Because of its extended half‐life (26 h) and strong affinity for the 5‐HT_1B/D_‐receptor, frovatriptan should be considered as a short‐term prophylactic treatment for chronic cluster headache [68]. Based on clinical experience, further research is recommended to assess the potential of frovatriptan as potential transitional therapy in episodic cluster headache or as potential (add‐on) preventative therapy for chronic cluster headache, without the need to withhold abortive therapy [69, 70, 71].

#### 4.1.3 Preventative/Prophylactic Treatment

Preventive therapy aims to prevent cluster headache attacks. However, because of the possibility of serious or limiting side effects of the medication, the dosage is usually increased gradually. To increase the likelihood of faster symptom relief, preventive strategies can be combined with transitional therapy.

##### 4.1.3.1 Verapamil

Verapamil is the preferred preventive treatment for cluster headache, although it is off‐label in some countries. Typically, treatment is started at a dosage of 40–120 mg administered three times daily, slowly increasing to 720–960 mg per day as needed [72]. Given its classification as a calcium channel blocker, increasing the dosage may lead to a spectrum of cardiac side effects, ranging from mild to severe. Therefore, it is strongly recommended that an electrocardiogram (ECG) be taken before starting the drug and that monitoring be continued during therapy [73, 74, 75]. Slow tapering of verapamil is advised to prevent cardiac complications when discontinuation is indicated. In episodic cluster headache, discontinuation of verapamil after an episode increases effectiveness during the next episode. Continuing verapamil outside of an episode may result in reduced or even loss of therapeutic effect. For chronic cluster headaches, a two‐month drug holiday is recommended if the efficacy declines. During breaks in therapy, the use of a bridging treatment should be considered.

##### 4.1.3.2 Lithium

Lithium is an alternative for the prevention of cluster headache. The initial impetus for trialing lithium for cluster headaches was the shared cyclical nature with bipolar disorder. Despite its effectiveness as a prophylactic therapy, verapamil is preferred because of its more favorable side‐effect profile [76]. The narrow therapeutic index of lithium contributes to both acute and long‐term side effects, including nausea, dizziness, and tremor. The optimal effect is achieved with a serum concentration between 0.7 and 1.2 mEq/L. [77] Because of its potential toxicity, monitoring hepatic, kidney, and thyroid function is required in addition to plasma concentration.

##### 4.1.3.3 Topiramate

Topiramate can be used as an alternative preventive therapy when verapamil or lithium is ineffective or not tolerated, but scientific support is limited. The starting dose is typically 50–100 mg twice per day, which may be increased to a maximum of 400 mg daily [78]. Gradual dosage titration (e.g., 25 mg/week) may decrease the incidence of common side effects such as cognitive disturbances, paresthesia, and weight gain [79]. Topiramate is particularly contraindicated in patients with nephrolithiasis and glaucoma.

##### 4.1.3.4 CGRP Antagonists

CGRP is a neuropeptide that is released in response to stimulation of the trigeminal nerve and binds to receptors in the vasculature and trigeminal system [80]. Elevated CGRP levels have been reported in the serum and tears of cluster headache patients [81, 82, 83, 84]. In episodic cluster headache, serum CGRP levels show a significant increase during attacks compared to the remission period, making it a potential target for preventive treatment. Consequently, CGRP antibodies are believed to play a multifaceted role in the pathophysiology of cluster headache, and further research is needed to elucidate this [85].

Galcanezumab is an anti‐CGRP monoclonal antibody administered by subcutaneous injection in doses of 240 to 300 mg monthly that has been shown to be effective in the treatment of episodic cluster headache. Access to this drug is limited due to the fact that galcanezumab is only approved in the United States [86, 87]. Although efficacy for chronic cluster headache has not been established in clinical trials, recent open‐label studies suggest that off‐label use could be beneficial for medically intractable cases [88, 89, 90, 91].

##### 4.1.3.5 Others

For the drugs listed below, the evidence is limited, and treatment can be rendered empirically based on comorbidities and expected side effects.

Melatonin supplementation as a preventive treatment is based on the circadian nature of cluster headaches and the observed low melatonin levels during and between bouts in patients with episodic cluster headaches [92, 93]. Although a small randomized controlled trial demonstrated a significant effect for patients with episodic cluster headache, subsequent studies were unable to replicate these findings when melatonin was used as an adjunctive therapy in addition to standard treatments [94, 95]. For chronic cluster headache patients, the effect was marginal [94, 96].

Gabapentin, an antiepileptic drug, is administered daily in tablet or capsule form at a total dose ranging between 900 and 3600 mg per day. Open‐label studies have shown a beneficial effect on attack frequency and duration in both episodic and chronic refractory cluster headache [97, 98, 99]. However, randomized controlled trials have yet to be conducted.

### 4.2 Interventional Management

**FIGURE 1:**
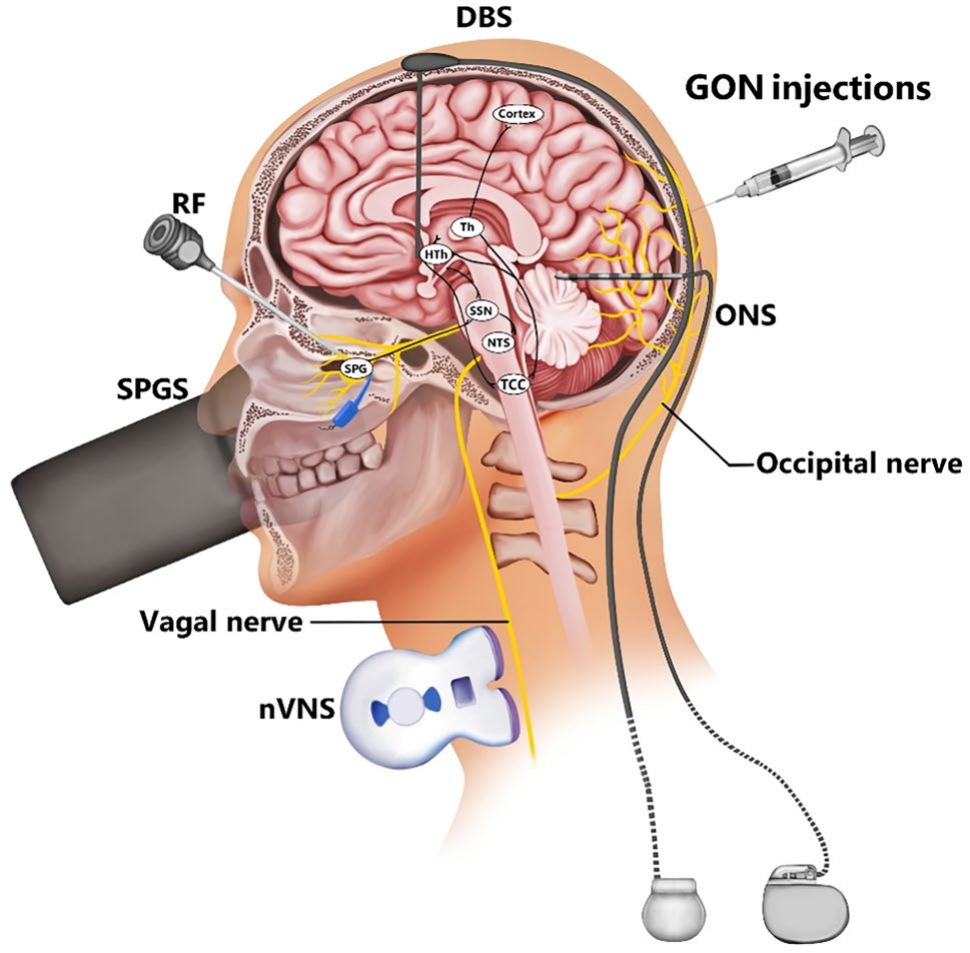
Schematic overview of the sites of action on neural pathways for interventional treatments for cluster headache. DBS = deep brain stimulation; GON‐injections = greater occipital nerve injections; HTh = hypothalamus NTS = nucleus tractus solitarius; nVNS = non‐invasive vagus nerve stimulation; ONS = occipital nerve stimulation; RF = radiofrequency therapy; SPG = sphenopalatine ganglion; SPGS = sphenopalatine ganglion stimulation; SSN = superior salivatory nucleus; TCC = trigeminocervical complex; Th = thalamus.

An interventional approach is indicated when pharmacological therapies are unsuccessful, particularly in the case of chronic cluster headache. In patients with MICCH, interventional approaches may be an effective treatment option. These approaches can be classified into one of three categories: abortive, transitional, and/or prophylactic. Figure 1 illustrates the current range of interventional treatments and their respective target areas.

#### 4.2.1 Greater Occipital Nerve Injections

Greater occipital nerve (GON) blocks are performed on the ipsilateral side of the pain using corticosteroids such as methylprednisolone and dexamethasone administered subcutaneously with a 25‐gauge needle [100]. The optimal location for the injection is determined by locating specific anatomical landmarks, namely the point located one‐third the distance between an imaginary line drawn between the external occipital protuberance and the mastoid process. Topical anesthetic cream may be applied to anesthetize the injection site. The co‐administration of a local anesthetic, such as lidocaine 2% (1 mL) and bupivacaine 0.5% (1 mL), with corticosteroid is a common practice because it serves a dual purpose: as a diagnostic or prognostic tool and to enhance pain relief [100]. When performing blocks using landmark guidance, paresthesia can be used as a guide. The potential benefits of imaging techniques such as ultrasound in reducing technical failure, like missing the nerve(s), have been recommended in guidelines but require further investigation. Although not formally studied in cluster headache, randomized controlled trials demonstrating efficacy for other headache disorders have also targeted the lesser occipital nerve(s) when there is tenderness over the nerve course or pain in its distribution. Further research is required to demonstrate the additional benefits of this approach in the context of cluster headache.

The following conditions constitute absolute contraindications in the procedure: allergy to anesthetics, an open skull defect or wound, infection at the procedure site, and patient refusal [100]. Administration of a GON injection may be contraindicated in patients who cannot remain still in the prone position, with coagulopathy, or who have a type I Arnold‐Chiari malformation [101, 102]. Described side effects immediately after injection include dizziness, blurred vision, and injection site sensitivity. Long‐term side effects after injections are rare, but altered hair growth or local atrophy have been documented in the literature, particularly when particulate steroids are used [103, 104]. There have been reports of transient side shift in cluster headache attacks following unilateral GON injections, though data on bilateral injections is limited [105, 106, 107].

Although the precise mechanism remains unclear, GON injection is a proven effective and safe monotherapy or add‐on therapy for both episodic and chronic cluster headaches [108, 109]. The effects of the injection(s) may persist for weeks to several months. Given the temporary effect, a single GON or greater and lesser ON injection can be an effective abortive treatment at the onset of a bout or transitional therapy in episodic cluster headache.

In practice, because of the effectiveness and low risk of side effects of GON injections, the procedure is often used as an effective and safe preventative treatment for chronic cluster headache, although the evidence for this indication is sparce [106]. The administration frequency is usually set at intervals of 3 months or longer, depending on the patient's response. However, there is currently no literature to support a standardized interval [100].

#### 4.2.2 Radiofrequency Therapy

The SPG, also referred to as the pterygopalatine ganglion (PPG), is located in the pterygopalatine fossa, posterior to the maxillary sinus. The ganglion contains the largest parasympathetic ganglion, postganglionic sympathetic fibers, and somatic branches arising from the maxillary nerve. It is surrounded by several important anatomical structures, including the processus pterygoideus, the sphenoidal sinus, the perpendicular plate of the palatine bone, and the pterygomaxillary fissure lateral [110].

The primary goal of performing an SPG block with the possible addition of radiofrequency (RF) therapy is to interrupt ganglionic activity, ideally at the onset of a cluster headache bout. The rationale for SPG ablation is rooted in its ability to relieve the parasympathetic symptoms and pain that patients experience during cluster headache attacks [110].

Radiofrequency ablation (RFA) is a thermal intervention that induces a heat lesion by applying a continuous high‐frequency electrical current generated by an RFA system. The procedure is typically performed under fluoroscopic guidance, allowing access to the SPG via intranasal, suprazygomatic, and infrazygomatic approaches, with the infrazygomatic route being predominantly utilized for RFA [111]. Case studies have also explored the use of CT for imaging, which may optimize treatment outcomes [112, 113]. Contraindications to RFA include the presence of local or systemic infections, coagulopathy, lack of even a temporary response to a prior (prognostic) SPG block, and hemodynamic instability. Caution is also advised in significant anatomical abnormalities, with restraint in performing the procedure.

For the procedure, a blunt 22‐gauge, 10 cm RFA needle with a 5–10mm active tip is used to deliver the electrical current, usually for a duration of 60 to 120 s, resulting in tissue temperatures of 80°C–90°C. This method has been shown to be effective primarily in episodic cluster headaches, but can also alleviate pain in (intractable) chronic cluster headaches [111, 113, 114, 115, 116].

However, RFA is not without risks. Documented complications include postoperative epistaxis, hemorrhage in the jaw, and unintended partial lesions of the maxillary nerve [111]. To reduce these risks, pulsed radiofrequency (PRF) can be used and has been shown to provide comparable benefit to RF, but more comparative‐effectiveness studies are needed [117]. Although the procedural approach remains the same, PRF delivers intermittent, short bursts of high‐voltage currents for up to 10 min, with tissue temperatures no higher than 45°C. The lower temperature in PRF offers a potentially safer alternative, significantly reducing the risk of side effects, including deafferentation symptoms and neuroma formation [118, 119, 120, 121]. Whereas these options may prove to be effective treatments for chronic cluster headache, larger and more robust studies are needed to confirm this.

Low‐temperature plasma radiofrequency ablation (LTPRA) has also been investigated in patients who have not responded to previous treatments, with encouraging findings [122]. Additionally, PRF treatment of the cervical roots at levels C1 and C2 has been described, though its effectiveness seems limited [123, 124].

#### 4.2.3 Neuromodulation Therapy

Over the past decade, there has been a significant increase in the use of neuromodulation techniques for cluster headache. These approaches become relevant when pharmacological and less invasive procedural interventions prove ineffective, indicating a refractory condition. Neuromodulation addresses both episodic and chronic cluster headaches and includes both invasive and non‐invasive modalities.

##### 4.2.3.1 Vagus Nerve Stimulation

Non‐invasive vagus nerve stimulation (nVNS) involves transcutaneous stimulation of the cervical portion of the ipsilateral vagus nerve. This technique is recommended as a well‐tolerated and effective abortive treatment for episodic cluster headache [125]. In addition, nVNS can be combined with standard care and serve as an adjunctive preventive treatment, aiming to reduce the frequency of attacks in patients with chronic cluster headache. This approach may also be beneficial for the subgroup of intractable patients [126, 127].

For nVNS, the portable device generates a low‐voltage electrical signal consisting of a series of 5 kHz sine waves, delivered over a duration of 1 ms, occurring every 40 ms. The device can deliver a maximum output of 24 V and 60 mA [126]. The abortive nVNS treatment protocol consists of three 2‐minute stimulation sessions, administered at five‐minute intervals, started as soon as possible after the onset of an attack. If the attacks persist longer than 9 min after the first session, the patient has the option to self‐administer up to three additional stimulation sessions. The preventive nVNS protocol consists of the administration of three stimulation sessions twice daily: the first dose is administered within an hour of waking up, with the second dose occurring 7 to 10 h later [126].

To the best of our knowledge, no absolute contraindications have been identified for nVNS, making it a viable option for patients who do not tolerate standard pharmacological treatments or for whom such treatments are contraindicated. Nevertheless, it is important to consider relative contraindications such as infections, anatomical abnormalities or alterations, and the presence of implanted devices in the neck area. Side effects of the device are infrequent and generally mild. Lip or facial drooping, pulling, or twitching, as well as irritation of the application site, paresthesia, and skin irritation, have been described. No serious device‐related side events have been reported [125, 126].

##### 4.2.3.2 Sphenopalatine Ganglion Stimulation

Sphenopalatine ganglion stimulation (SPGS) is an invasive treatment that relies on the surgical implantation of a neurostimulator in the pterygopalatine fossa, the cavity in which the largest parasympathetic ganglion is located. The rationale for neurostimulation is based on the involvement of the SPG in the trigeminal parasympathetic reflex, which is responsible for the autonomic symptoms observed in cluster headache [128, 129]. Although the exact mechanisms are not fully understood, the stimulation of the SPG leads to a reduction in parasympathetic outflow that interferes with the trigeminal parasympathetic reflex [130].

Given the inherent variability of midface anatomy both within and between patients, it is strongly recommended that preoperative CT imaging be performed to ensure accurate placement. The microstimulator, consisting of a lead with six electrodes and a fixation plate, is implanted under general anesthesia via a buccal incision near the molars and secured to the zygomatic process of the maxilla. After surgery, the patient can charge and operate the device with a remote controller that uses induction [131].

Currently, only the efficacy of abortive treatment in chronic cluster headache has been demonstrated in a small sham‐controlled study and proven to be a long‐term solution [132, 133, 134]. A possible prophylactic effect, resulting in a reduction in attack frequency, was observed in 35% of patients, while conversely, an increase in attack frequency was reported in one‐third of the cohort [133].

Relative contraindications for this procedure include the presence of a local infection in the surgical area, a history of (iatrogenic) osteoporosis, and previous skull base surgeries in the patient's medical history [131]. There is no limit to the number of stimulation sessions, and no cardiovascular or systemic side effects have been reported. Most documented side effects are attributable to the surgery itself, including sensory disturbances, postoperative pain, and swelling [132]. Battery capacity is not specified in the literature. However, at the time of writing, SPG treatment is not widely available, as there are currently no certified SPG stimulation systems.

##### 4.2.3.3 Occipital Nerve Stimulation

Occipital nerve stimulation (ONS) is a minimally invasive neuromodulation therapy which delivers electrical pulses to the greater and lesser occipital nerves. The GON, a medial branch of the dorsal ramus of the C2 spinal nerve, passes through the inferior oblique muscle, pierces the semispinalis capitis, and then the aponeurotic insertion of the trapezius and sternocleidomastoid muscles. This purely sensory nerve innervates the skin of the posterior skull, including the temporal regions. The lesser occipital nerve branches from both C2 and C3 spinal roots and provides sensory innervation to the posterior part of the skin of the auricle [135]. Both nerves influence second‐order nociceptors in the brainstem that play a role in pain perception [136].

ONS involves subcutaneous implantation of either a single 8‐contact cylindrical lead or two 4‐contact cylindrical leads, based on the chosen lateral or medial approach. The leads are placed via a 14‐gauge Tuohy needle at the level of the external occipital protuberance, targeting both GONs. This bilateral targeting addresses the documented potential for cluster headaches to shift sides [137, 138, 139]. The leads are tunneled and connected to an implantable pulse generator (IPG), typically located in the gluteal or abdominal region [140].

To reduce infection risk, the occipital area from the top of the ear line down to the neck should be shaved before surgery, and antibiotic prophylaxis is administered during the procedure. A postoperative skull x‐ray confirms lead placement. Relative contraindications for the implantation procedure include electrical devices (e.g., pacemakers, spinal cord stimulators) that could be affected by or affect stimulation, Arnold‐Chiari malformation, pregnancy, local infections, and bleeding disorders [141].

During follow‐up, stimulation settings are optimized to ensure paresthesia within the GON distribution. Standard parameters include tonic stimulation with biphasic square waves, with a pulse width of 200–500 µs and frequencies of 10–40 Hz. Amplitude is adjusted based on patient comfort and perception [142]. However, no specific parameters consistently predict treatment success [140]. While paresthesia guides adjustments, its role in efficacy is unclear and can be uncomfortable. Burst stimulation, which does not induce paresthesia, has shown promising results in small studies, offering the potential for future blinded research [143, 144, 145].

Early studies demonstrated that ONS reduces the attack frequency by more than 50% in at least half of patients. However, adverse events related to the hardware are common, with battery depletion being the most frequent issue [140, 146, 147, 148]. Rechargeable batteries and lower current intensities could help mitigate battery depletion issues viewed by some as complications.

Initial off‐label use of spinal cord stimulation (SCS) electrode leads to stimulate the occipital nerve contributed to high rates of lead breakage, lead migration, and infections from pressure ulcers [139, 140, 147, 148, 149]. The use of paddle leads and the introduction of dedicated ONS leads with silicone tines has possibly reduced lead migration. Although no consensus exists on optimal surgical techniques or lead positioning, recent refinements have reduced complications and improved outcomes. Since it can take 2 to 10 months for patients to be classified as responders (usually defined as a 50% or greater reduction in symptoms, though smaller reductions are widely considered to be clinically meaningful), ONS systems are typically implanted without a trial period [137, 150]. Despite the early challenges, large‐scale studies now confirm that ONS is both effective and durable over time [139, 142, 148, 151].

##### 4.2.3.4 Deep Brain Stimulation

Deep brain stimulation (DBS) is a neuromodulation technique that can target different brain regions depending on the clinical indication. In the case of cluster headache, the target is the hypothalamus, a structure located at the base of the brain, just above the pituitary gland and below the thalamus, adjacent to the third ventricle. The hypothalamus is integral to the regulation of various circadian and homeostatic functions, such as sleep and hormone secretion. Given the role of the hypothalamus in circadian rhythm regulation, it has been identified as a potential target for the treatment of cluster headache, which often present with circadian symptomatology. Functional brain imaging also shows increased activity in the hypothalamus in cluster headache patients, further supporting the relevance of the hypothalamus in the pathophysiology of cluster headache [152, 153].

Over the years, various surgical protocols have emerged, differing in target regions, anesthesia type, and technique. The following outlines the general approach. Preoperative whole‐brain MRI stereotaxy is performed to map the trajectory for electrode placement, combined with a T2‐weighted fast spin echo sequence with 2 mm slice thickness for high‐resolution visualization of critical landmarks like the hypothalamic commissures [154]. Although no consensus exists on the exact DBS target for MICCH, zones of interest include the posterior‐inferior and ventroposterior hypothalamic regions, ventral tegmental area, and mammilotegmental fasciculus, with the latter showing the most promising results [155, 156, 157].

The surgery can be performed under sedation or general anesthesia. Microelectrode recording (MER) is used to guide lead placement by monitoring neural activity through a burr hole. Once the target is confirmed, the permanent DBS lead is implanted. Intraoperative macrostimulation is conducted to assess side effects (e.g., diplopia, dizziness, vertigo) and ensure accurate lead placement. MER and test stimulation are typically performed without sedation. After the lead is secured to the skull, general anesthesia is usually induced to tunnel the lead extender to the pulse generator, placed in a subclavicular pocket [154].

Postoperative MRI is used to confirm electrode placement, with the closest contact to the target selected for stimulation. The device is activated in monopolar mode with a pulse width of 60 μs and a frequency of 180 Hz. Voltage is increased gradually during follow‐up to a maximum of 2.0 V, or just below the threshold for side effects. Therapeutic effects may take days to months to develop [154, 157]. Typically, only one lead is implanted, as contralateral episodes of chronic cluster headache occur in only about 3% of patients [157].

A number of open‐label studies have demonstrated a favorable response to DBS in patients with MICCH. A recent meta‐analysis reported a reduction in the average headache attack frequency of 70.1% and a 50.6% decrease in headache intensity after DBS [157]. Another meta‐analysis found a 77% pooled responder rate in MICCH patients treated with DBS [156]. Typically, DBS is considered after patients have failed less invasive modalities such as SPGS or ONS. Although the one randomized controlled trial, a crossover study in 11 patients with chronic cluster headache, reported no improvement with 3 serious complications (subcutaneous infection, micturition syncope, and transient loss of consciousness), 6 of 11 patients in the 12‐month open‐label follow‐up period responded with a > 50% reduction in attack frequency [158].

Similar to ONS, battery depletion occurs, with the most common reported complications including oculomotor disturbances, dizziness, changes in libido, and alterations in sensation [157]. Perioperative complications that have been reported include electrode misplacement or breakage, deep surgical site infection, and neurological deficits. The incidence of these complications is comparable to that seen in other indications for DBS. Notably, one patient experienced an intracerebral and intraventricular hemorrhage due to a panic attack during MER [159].

##### 4.2.3.5 Others

Ongoing research continues to explore new treatment modalities. Despite the favorable outcomes with (pulsed) RF of the SPG, the effects tend to be temporary, requiring repeated interventions. As a result, the potential of Gamma Knife radiosurgery is also being investigated; however, the outcomes have been variable thus far, with therapeutic effects that also tend to diminish over time [160, 161].

The use of tonic spinal cord stimulation (SCS) as a preventive treatment has also been explored in neuromodulation‐naive patients. The concept is that high cervical stimulation may deliver electrical pulses more effectively to the trigeminocervical complex than ONS [162]. Additionally, one retrospective study reported effectiveness using high‐frequency SCS without paresthesia in a wide variety of headache disorders, including cluster headache. However, as a retrospective study, it lacks the ability to adequately assess treatment efficacy [163].

## 5 Recommendations

### 5.1 Clinical Practice Algorithm

**FIGURE 2:**
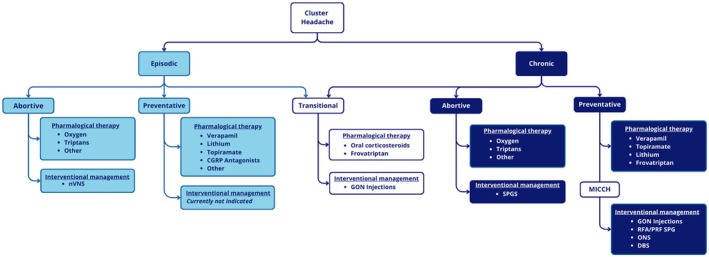
Clinical practice algorithm of treatment strategies for cluster headache in chronological order.

The treatment of cluster headache remains challenging due to the wide range of potential therapies, many of which are backed by limited evidence, and an incomplete understanding of its pathophysiology. No single treatment is universally effective. In clinical practice, some treatment modalities can be used concurrently; for instance, a good responder to ONS therapy may still require abortive pharmacological treatments, such as triptan injections or high‐flow oxygen, as attacks may continue to occur. This guideline provides a structured overview of treatment options, categorized by the form of cluster headache and treatment modality, summarized in a flow diagram (see Figure 2). Although the literature does not establish a clear hierarchical approach, we propose a chronological sequence for offering these treatments to patients.

Although the few comparative studies conducted thus far deserve attention, they should be interpreted with caution. In the context of acute treatment, a network meta‐analysis indicates that high‐flow oxygen therapy (at least 12 L/min) and sumatriptan injections (6 mg subcutaneously) are more effective than low‐flow oxygen, zolmitriptan (10 mg nasal spray), octreotide, and nVNS [164]. Furthermore, a prospective self‐reported outcome study also indicates that sumatriptan is perceived by patients as more effective [165]. Oxygen is considered the most effective treatment for episodic cluster headaches, while sumatriptan is the preferred option for chronic cluster headache [165].

For transitional and/or prophylactic treatment, a retrospective comparator study indicates that oral steroids provide a superior short‐term prophylactic response than GON injections [166]. However, it should be noted that oral steroids are associated with an increased risk of long‐term side effects. Regarding the effectiveness of RFA and PRF, both techniques have been shown to be equally effective. However, PRF is preferable because of its more favorable side‐effect profile [117].

### 5.2 Management of Cluster Headache in Obstetric Patients

Women develop cluster headache at an earlier age than men, and recent estimates suggest a smaller male‐to‐female ratio than was previously reported. As the age of pregnant women continues to increase, it is likely that treating obstetric patients with cluster headaches will become more common [4, 5]. Although remission of cluster headaches during pregnancy has been reported, managing the condition can be challenging [4, 167]. Patients should be informed about the off‐label use of most treatments and the limited evidence for efficacy and safety during pregnancy and lactation [168, 169]. Given the complexity of treating debilitating chronic pain during pregnancy, it is recommended that the patient be managed by a multidisciplinary team consisting of at least a headache specialist and an experienced gynecologist within a specialized headache center [168, 170].

In the case of abortive therapy, oxygen therapy is the optimal first‐line treatment because it has no adverse effects on the mother or child during the critical periods of pregnancy and lactation. As for sumatriptan, no increased risk of congenital anomalies has been identified, and its use during lactation does not appear to have any adverse effects [168, 169]. However, there are no data on the safety of repeated daily doses, which are often required for cluster headache. Therefore, daily use is not recommended. The use of ergotamine derivatives is contraindicated for the treatment of cluster headaches during pregnancy because of the potential impact on fetal blood supply [170]. The available literature on lidocaine nasal spray and somatostatin analogues is limited. When used as a local anesthetic for nerve blocks, lidocaine is considered safe during pregnancy and lactation [171]. In animals, systemic lidocaine (i.e., infusions) has been associated with behavioral abnormalities in offspring [172]. Caution should be exercised regarding the use of somatostatin analogues, and close monitoring of the child is recommended [169].

Ketamine use during gestation has been associated with neurocognitive effects, and its use should be limited to abortive therapy as a rescue drug [173, 174, 175]. Short‐term use of corticosteroids for transitional therapy may help and is unlikely to result in significant side effects. However, caution is advised, especially during the first trimester, because of an established association with cleft lip and palate. It is currently unclear whether GON injections offer a superior safety profile compared to oral steroids [171].

The number of pharmacologic preventive options available during pregnancy is limited. Verapamil is considered a safe drug with respect to congenital anomalies, although perinatal complications have been documented at doses lower than those commonly used to treat cluster headaches [169, 171]. Topiramate and lithium are contraindicated because of the potential for adverse maternal and fetal effects. If treatment is unavoidable, close monitoring of serum levels and early echocardiography are recommended [171]. Given the limited real‐life data on galcanezumab, it is advisable to refrain from its use during pregnancy as a precautionary measure.

To the best of our knowledge, there is only one case report in the literature on the use of interventional treatment for cluster headache during pregnancy for ONS. The patient remained attack‐free until 35 weeks of pregnancy when the headache returned, purportedly due to incorrectly recharging the battery. However, after properly recharging the battery, the headache attacks persisted. When the battery was recharged immediately postpartum, the headache attacks gradually decreased over the course of 4 weeks [176].

We do not recommend elective surgery during pregnancy, as procedures such as RF therapy or neuromodulation implantation involve radiation exposure, posing risks to the fetus. However, the use of neurostimulators during pregnancy appears to be safe. To date, no adverse effects related to neurostimulation during pregnancy have been reported. Given the limited available literature, we recommend closer patient monitoring if there is an increase in symptoms or nonspecific complaints during pregnancy.

## 6 Summary

Cluster headache is a disabling condition, especially in the chronic form. Evidence for treatment is generally limited to off‐label use and expert opinion. The only well‐established treatments are oxygen therapy and sumatriptan. For preventive care, verapamil and CGRP antagonists show promise, with the latter being a first mechanism‐based treatment option.

Pharmacological treatments tend to be effective in episodic cluster headaches but less so in chronic forms, potentially leading to MICCH. Although structures such as the hypothalamus, SPG, and trigeminocervical complex are implicated in the disease's pathophysiology and serve as targets for invasive treatments, the precise mechanisms remain unknown.

Many interventional treatments, such as GON injections and PFR treatments, offer only temporary relief. Abortive neurostimulation therapies such as SPGS and nVNS may be effective, though evidence and device availability are limited. Preventive neurostimulation treatments, such as ONS, appear suitable for MICCH patients. DBS could be considered as a last‐resort option, requiring stringent patient selection.

Despite the expanding range of treatment options for cluster headache, evidence remains limited. Large, long‐term studies are needed to assess the efficacy and durability of these treatments.

## Author Contributions

Casper S. Lansbergen performed the literature search and wrote the manuscript. Cecile C. de Vos, Rolf Fronczek, and Frank J.P.M. Huygen assisted in the selection of the literature and revised the manuscript. Frank J.P.M. Huygen was held responsible for this manuscript. Leopoldine A. Wilbrink and Steven P. Cohen revised and edited the manuscript.

## Ethics Statement

The study was conducted according to the principles of the Declaration of Helsinki.

## Consent

As there were no new data or patients submitted to this study, consent from patients is not required in the publication of this article.

## Conflicts of Interest

Frank J.P.M. Huygen is an Editorial Board member of Pain Practice and a co‐author of this article. To minimize bias, this author was excluded from all editorial decision‐making related to the acceptance of this article for publication. The other authors declare no conflicts of interest.

## Supporting information


Appendix S1.

## Data Availability

Data sharing is not applicable to this article as no new data were created or analyzed in this study.
