# Supplementary material for: 15. Cluster Headache
Source: Pain Pract. 2025 May 28;25(5):e70050. doi: 10.1111/papr.70050 (PMC12120220; doi:10.1111/papr.70050)
Supplement: Supplementary file 1 — Appendix S1. [file PAPR-25-0-s001.docx]

**Appendix 1. Overview of the scientific literature per interventional treatment for cluster headache, including quality assessment and main results.**

| **Intervention** | **Authors** | **Year** | **Study design** | **Studies** | **Quality assesement** | **Main results** |
| --- | --- | --- | --- | --- | --- | --- |
| **GON-injections** | Gordon A *et al.* | 2023 | Systematic Review | 2 randomized controlled trials | *Randomized controlled trials:* One study had a high risk of selective reporting bias, while the other had a low risk of bias. | **Efficacy**: GON injections effectively reduces CH attack frequency, severity, and duration, with higher response rates when combined with long-term preventive medication. |
|  |  |  |  | 8 prospective cohort studies | *Open-label studies*: Generally poor quality with high risk of confounding, selection bias, and inconsistent reporting. | **Safety**: Generally safe, with mostly transient adverse effects; methylprednisolone may have a better safety profile, but data on adverse effects remain unreliable. |
|  |  |  |  | 8 retrospective cohort studies | *Case reports/series*: High risk of selection bias, poor reporting consistency, and limited reproducibility. | **Clinical implications**: GON injections are best used as transitional therapy, as a high frequency of injections may be associated with side effects. |
| **Radiofrequency therapy** | Jain E *et al.* | 2021 | Systematic Review | 5 prospective cohort studies | *Prospective cohort studies*: Moderate quality, but potential bias due to lack of randomization. | **Efficacy:** RFA reduces pain scores and provides both short- and long-term relief, but variability in techniques and targets limits generalizability. |
|  |  |  |  | 5 retrospective cohort studies | *Retrospective studies*: Higher risk of bias, with issues in data collection and confounding factors. | **Safety:** No major adverse events reported, but long-term safety data are limited due to the retrospective nature of most studies |
|  |  |  |  | 1 case series and 1 case report | *Case series & case report*: Low-quality evidence with high risk of selection bias and poor generalizability. | **Clinical Implications:** RFA may be considered for refractory chronic cluster headache, but standardized treatment protocols are lacking. |
| **Non-invasive vagus nerve stimulation** | Fernandez-Hernando D *et al.* | 2023 | Systematic Review | 3 randomized controlled trials | *Randomized controlled trials:* The RCTs in the review showed moderate-to-high quality, with two studies having a low risk of bias and one showing some concerns. | **Efficacy:** Moderate-to-high-quality evidence suggests nVNS reduces the frequency and intensity of episodic cluster headache, but shows limited effectiveness for chronic cluster headache. It may be beneficial when added to standard of care for chronic cluster headache. |
|  |  |  |  |  | The studies had good methodological quality (scores between 6 and 8 out of 10), but moderate risk of publication bias was noted. | **Safety:** No major adverse events were reported in most studies. One study reported 59 side effects, including 35 serious adverse events, primarily hardware-related, but no deaths occurred. |
|  |  |  |  |  |  | **Clinical Implications:** nVNS shows promise for treating episodic and chronic cluster headache with fewer side effects, but further studies with standardized protocols are needed to confirm its efficacy and safety. |
|  | de Coo IF *et al.* | 2019 | Meta-Analysis | 2 randomized controlled trials | *Randomized controlled trials:* Moderate quality due to the small sample size in both studies and differences in trial design (e.g., treatment duration, extra pulses in ACT2), though the trials provide controlled data with significant findings for episodic cluster headache. | **Efficacy**: nVNS was effective for aborting attacks in episodic cluster headache but showed limited efficacy for chronic cluster headache.  **Safety**: nVNS was well tolerated with no significant safety concerns, and adverse events were mild.  **Clinical Implications**: nVNS offers practical advantages over current treatments, including ease of use, flexibility for frequent treatments, and safety for patients with cardiovascular contraindications. |

| **Sphenopalatine ganglion stimulation** | Rosso C. *et al.* | 2019 | Systematic Review | 1 randomized controlled trail | *Randomized controlled trial:* Moderate quality due to a small sample size (28 participants) and a short follow-up period (12 months). | **Efficacy**: SPG stimulation results in a significant reduction in the frequency of episodic cluster headache, but its effectiveness for chronic cluster headache still needs to be proven. |
| --- | --- | --- | --- | --- | --- | --- |
|  |  |  |  | 1 prospective cohort studies | *Cohort study*: Moderate quality, with a follow-up of 24 months; 35.4% of patients had positive outcomes, but variability in results and lack of randomization limit generalizability. | **Safety**: Common side effects include temporary facial paresthesia, pain, and swelling post-implantation, with some cases of infections and paresis. |
|  |  |  |  | 2 case series | *Case series:* Moderate quality due to variability in outcomes across studies. | **Clinical implications:** SPG neurostimulation is effective for episodic cluster headache as an abortive treatment, but its effectiveness for preventive treatment in cluster headache has not yet been proven. |
| **Occipital nerve stimulation** | Membrilla et al. | 2022 | Systematic Review | 1 randomized controlled trial | *Randomized controlled trials:* The RCT had low risk of bias and was an electrical dose-controlled study. | **Efficacy:** ONS shows sustained effectiveness for refractory chronic cluster headache, with response rates over 50%. |
|  |  |  | and Meta-Analysis | 3 prospective cohort studies | *Prospective cohort studies*: Moderate quality, but potential bias due to lack of randomization. | **Safety**: Most adverse events are related to hardware malfunction and technical complications, but these are expected to decrease with device advancements. |
|  |  |  |  | 1 retrospective cohort study | *Retrospective cohort study:* Higher risk of bias, due to missing data. | **Clinical Implications**: ONS is a cost-effective therapy that may reduce the need for medications. However, its availability is limited to specialized centers, and further high-quality studies are needed to strengthen the evidence. |
|  |  |  |  | 5 case series and 1 case report | *Case series and caser report*: Low-quality evidence with high risk of selection bias and poor generalizability. |  |
| **Deep brain stimulation** | Membrilla et al. | 2022 | Systematic Review | 1 randomized controlled trial | *Randomized controlled trials:* Moderate quality due to a small sample size and a failure to meet the primary endpoint, though it provides controlled data. | **Efficacy:** DBS shows high response rates (up to 100%) for hypothalamic stimulation with a pooled response rate of 77%. |
|  |  |  | and Meta-Analysis | 1 prospective controlled trial | *Prospective controlled trial*: Overall higher risk for bias, due to methodological bias directly related to the study design. | **Safety:** Adverse events primarily related to electrical dose adjustments; rare severe complications like hemorrhages. |
|  |  |  |  | 1 prospective cohort study | *Prospective cohort study*: with varied response rates across different stimulation targets. Overall, the outcomes suggest potential efficacy for DBS in chronic cluster headache. | **Clinical Implications**: DBS offers effective treatment but with surgery-related risks. Regarding the location, the mammillotegmental fasciculus target appears to offer the best outcomes, but further research is needed. |
|  |  |  |  | 7 case series | *Case series*: Low to moderate quality due to small samples and varying response rates. The mammillotegmental fasciculus showed strong results but was only studied in one small series. |  |
|  | Murray MM *et al.* | 2023 | Systematic Review | 1 randomized controlled trial, | *Randomized controlled trials:* Only one small pilot RCT was conducted with a small sample size. | **Efficacy:** While non-randomized studies suggest DBS significantly reduces headache frequency and intensity in most patients, the only RCT found no significant benefit, limiting the overall level of evidence. |
|  |  |  | and Meta-Analysis | 9 prospective trials | *Prospective controlled trial*: Low to moderate quality due to the lack of blinding or control groups. | **Safety:** DBS has a low mortality rate (<1%) but carries a notable risk of major complications (16.67%), including bleeding, particularly with microelectrode recording. |
|  |  |  |  | 2 retrospective trials | *Retrospective controlled trial*: Moderate quality due to potential bias from the retrospective design, small sample size, and variability in patient outcomes. | **Clinical implications:** DBS should be considered for refractory chronic cluster headache patients, with careful selection to optimize outcomes, and future advancements in lead technology may further improve efficacy and safety. |
|  |  |  |  | 4 case series | *Case series*: Majority of studies were prospective case series with a high risk of bias. |  |
